# Endocrine manifestations and long-term outcomes of patients with mitochondrial diseases

**DOI:** 10.1186/s13023-025-03773-6

**Published:** 2025-05-17

**Authors:** Ja Hye Kim, Dohyung Kim, Soojin Hwang, Gu-Hwan Kim, Beom Hee Lee, Han-Wook Yoo, Jin-Ho Choi

**Affiliations:** 1 Department of Pediatrics, Asan Medical Center, University of Ulsan College of Medicine, Seoul, Korea; 2 Medical Genetics Center, Asan Medical Cente, University of Ulsan College of Medicine, Seoul, Korea; 3 Department of Pediatrics, Bundang CHA Medical Center, CHA University, Seongnam, Korea

**Keywords:** Mitochondrial encephalomyopathy, lactic acidosis, stroke-like episodes, Pearson syndrome, Kearns–Sayre syndrome, Diabetes mellitus, Adrenal insufficiency, Hypoparathyroidism

## Abstract

**Background:**

Endocrine dysfunctions are commonly associated with mitochondrial diseases. This study aimed to investigate clinical characteristics and outcomes of endocrine manifestations in patients with mitochondrial diseases.

**Methods:**

This study included 54 patients from 47 families with mitochondrial diseases who were genetically confirmed; 49 patients with mitochondrial encephalomyopathy, lactic acidosis, and stroke-like episodes (MELAS), four with Pearson syndrome, and one with Kearns–Sayre syndrome (KSS). Clinical and endocrine findings were retrospectively reviewed.

**Results:**

The median age at diagnosis was 18.5 years (range, 0.1 − 49 years). In 49 patients with MELAS, the mean height and weight standard deviation scores were − 2.0 ± 1.3 and − 2.6 ± 1.6, respectively, with 44.9% (*n* = 22) of the patients exhibiting short stature at diagnosis. Twenty-three (46.9%) patients with MELAS were diagnosed with diabetes mellitus (DM) at a median age of 26 years (range, 12 − 50 years). Interestingly, papillary thyroid cancer was observed in 10.2% of patients (*n* = 5) with MELAS at a mean age of 34.1 ± 6.9 years. One patient with MELAS and one with KSS exhibited hypoparathyroidism. Patients with Pearson syndrome and KSS exhibited more severe short stature. Adrenal insufficiency was noted in 50% of the patients with Pearson syndrome.

**Conclusions:**

In 20% of patients with MELAS, endocrine dysfunctions including having a short stature, DM, and hypoparathyroidism preceded the onset of neurological manifestations. Papillary thyroid cancer occurred in 10% of patients with MELAS. Patients with Pearson syndrome and KSS showed profound growth retardation and multisystem dysfunctions, such as chronic kidney disease and neurological defects, which contributed to increased mortality.

**Supplementary Information:**

The online version contains supplementary material available at 10.1186/s13023-025-03773-6.

## Background

Mitochondrial diseases are genetically heterogeneous, multisystem disorders resulting from defects in the mitochondrial respiratory chain and oxidative phosphorylation system. These are the most commonly inherited metabolic diseases, occurring in approximately 1 in every 5,000 people worldwide [1, 2]. Mitochondria are dynamic cellular organelles that play an essential role in the production of adenosine triphosphate (ATP), the fundamental unit of cellular energy metabolism. They also play key roles in calcium homeostasis, programmed cell death, and the biosynthesis of steroid hormones from cholesterol [3].

Mitochondrial dysfunction can potentially affect any organ system because of the ubiquitous presence of mitochondria, with neurological manifestations being the most prominent [4]. However, certain mitochondrial disorders, such as Leber’s hereditary optic neuropathy (LHON), predominately affect a single organ — in this case, the eyes [1]. Mitochondrial diseases are classified based on clinical phenotypes and specific genetic mutations [5]. The number of mitochondria per cell varies considerably, ranging from hundreds to thousands, and these organelles are distributed randomly during cell division [6]. Variants in mitochondrial genes are not uniformly present in all copies of the gene; the percentage of these mutant genes is referred to as heteroplasmy [6]. Higher heteroplasmy levels generally result in more severe disease phenotypes. Consequently, patients with identical pathogenic variants can exhibit a wide spectrum of clinical features.

Endocrine dysfunctions are a significant manifestation of mitochondrial diseases, with varying incidence rates depending on specific mitochondrial DNA (mtDNA) mutations, degree of heteroplasmy, energy demands of the particular tissues, threshold effects, nuclear gene defects, and environmental factors [3]. The most prevalent endocrine dysfunction is diabetes mellitus (DM), which is a monogenic form of diabetes caused by mutations in mtDNA. The association of DM with mitochondrial diseases, referred to as maternally inherited diabetes mellitus and deafness (MIDD), is particularly well-documented in the *MTTL1* m.3243 A > G mutation, which is frequently associated with mitochondrial encephalomyopathy, lactic acidosis, and stroke-like episodes (MELAS). The molecular mechanism by which the m.3243 A > G mutation affects insulin secretion may involve the attenuation of cytosolic ADP/ATP levels, leading to a resetting of the glucose sensor in pancreatic β-cells [7]. Age-dependent deterioration of pancreatic function in carriers of the m.3243 A > G mutation is attributed to changes in ATP and reactive oxygen species levels [7]. Although uncommon in mitochondrial disease, hypoparathyroidism is a hallmark of Kearns–Sayre syndrome (KSS), which results from large-scale mtDNA rearrangements [1, 8]. Pearson syndrome is characterized by sideroblastic anemia, exocrine pancreatic insufficiency, insulin-dependent DM, and hypoparathyroidism in early childhood [9]. Short stature is commonly observed in these conditions, occurring in 20−50% of patients with MELAS and in 38−90% of those with Leigh syndrome and KSS [10–12]. Hypogonadism may result from hypothalamic dysfunction or end-organ defect, and premature ovarian failure has also been observed [13].

Various endocrine dysfunctions can occur in mitochondrial diseases; however, most studies are cross-sectional and lack long-term clinical data [10, 14]. Therefore, this study aimed to investigate the long-term course of endocrine dysfunctions in patients with mitochondrial diseases at a single academic center.

## Methods

### Patients

This study included 54 patients from 47 families with primary mitochondrial disease: 49 with MELAS, 4 with Pearson syndrome, and 1 with KSS, who were diagnosed between January 1998 and December 2023 at the Department of Pediatrics, Asan Medical Center, Seoul, Korea.

The patients were diagnosed with MELAS, KSS, or Pearson syndrome based on their clinical features and genetic testing. MELAS with symptoms occurring before the age of 18 years was categorized as juvenile-onset disease, whereas MELAS beginning at the age of ≥ 18 years was classified as adult-onset disease. KSS was defined by the following triad: onset before the age of 20 years, chronic progressive external ophthalmoplegia, and pigmentary retinopathy, with the diagnosis genetically confirmed by a large deletion in mtDNA [15]. Pearson syndrome was defined by the presence of bone marrow failure in the first year of life, followed by lactic acidosis, pancreatic insufficiency, renal tubulopathy, and failure to thrive [9]. The diagnosis of Pearson syndrome was confirmed by a single large deletion of mtDNA [9].

This study excluded patients with LHON, Myoclonic Epilepsy with Ragged Red Fibers, and Leigh syndrome because these are single-organ conditions, such as optic nerve dysfunction or neurological impairments, rather than endocrine abnormalities. This study was approved by the Institutional Review Board of the Asan Medical Center, Seoul, Korea (IRB No. 2024 − 0271). This study was exempt from the requirement of informed consent because of the retrospective nature of the study and the analysis used anonymous clinical data.

### Endocrine and molecular genetic investigations

Data from medical records including age, sex, height, weight, body mass index (BMI), family history of mitochondrial diseases, and laboratory findings including serum glucose, calcium, creatinine kinase, phosphorus, lactic acid, and hemoglobin A1c were retrospectively reviewed. Height and weight are expressed as standard deviation scores (SDSs) based on age- and sex-matched references [16]. Short stature was defined as a height SDS < − 2 according to normative data.

Serum thyroid-stimulating hormone (TSH) and free thyroxine (T4) levels were measured using an immunoradiometric assay (IRMA, TSH CTK-3^®^, DiaSorin, Saluggia, Italy) and radioimmunoassay (RIA, FT4 RIA Kit^®^, Beckman Coulter, Prague, Czech Republic), respectively. Intact parathyroid hormone (PTH) level was measured by IRMA (Shin Jin Medics Inc., Goyang, Korea). Serum insulin-like growth factor 1 (IGF-1) and IGF-binding protein 3 (IGFBP-3) levels were determined by IRMA (Immunotech, Marseilles, France).

Genomic DNA was extracted from peripheral blood leukocytes using a Puregene DNA isolation kit (Qiagen, Hilden, Germany). To identify single nucleotide substitutions and small insertions/deletions, mtDNA was amplified by polymerase chain reaction (PCR) using 24 pairs of specific oligonucleotide probes (Table S1). After amplification, the PCR products were directly sequenced using the BigDye Terminator V3.1 Cycle Sequencing Kit (Applied Biosystems, Foster City, CA, USA) according to manufacturer’s instructions. To detect the large deletions, mtDNA was amplified using a long-distance PCR with various primer combinations, as previously described [17]. Seven regions of the mtDNA were targeted for detecting deletions, and the primers were specifically designed to optimally identify mtDNA deletions of various sizes (Table S2). After amplification, the PCR products were separated on an agarose gel and then directly sequenced. This was performed using the same primers to cover the deletion regions with the BigDye Terminator V3.1 Cycle Sequencing Kit (Applied Biosystems, Foster City, CA, USA). Electrophoresis and subsequent analysis of the sequencing reactions were conducted using an ABI 3130 Genetic Analyzer (Applied Biosystems, Foster City, CA, USA).

## Results

### Clinical characteristics at presentation

The median age at presentation was 18.5 years (range, 0.1 − 49 years, Table 1), with a median follow-up duration of 11 years (range, 2 − 17 years). Of the 49 patients from 42 families with MELAS, 21 patients (42.9%) from 18 families were classified as having juvenile-onset disease, with a median age of 11 years (range, 0.1 − 17 years). Among them, 18 patients (85.7%) presented with neurological symptoms including developmental delay (*n* = 7), seizures (*n* = 9), and stroke-like episodes (*n* = 2). Two patients (9.5%) presented with cardiomyopathy, and one (4.8%) with rapidly progressive glomerulonephritis. Among the 28 patients (57.1%) from 27 families with adult-onset disease, the median age at presentation was 28 years (range, 18 − 49 years). Of these, 14 patients (50%) presented with neurological symptoms, 10 (35.7%) with DM, 2 (7.1%) with cardiomyopathy, 1 (3.6%) with proteinuria, and 1 (3.6%) with severe fatty liver. Sensorineural hearing loss (SNHL) was observed in 17 patients (81%) with juvenile-onset disease and 21 (75%) with adult-onset disease. As the precise age of onset of SNHL could not be determined, it was excluded from the analysis of initial presentation.

**Table 1:** Clinical characteristics of patients with mitochondrial diseases

		MELAS			Pearson syndrome	Kearn–Sayre syndrome
		Total	Juvenile	Adult		
Number (female: male)		49 (29:20)	21 (9:12)	28 (19:9)	4 (1:3)	1 (0:1)
Median age at presentation, years (range)		18.5 (0.1, 49)	11 (0.1, 17)	28 (18, 49)	0.3 (0.1, 2.8)	0.1
Presenting features	Intellectual disability	8 (16.3%)	7 (33.3%)	1 (3.6%)	0	0
	Stroke-like episode	6 (12.3%)	2 (9.5%)	4 (14.3%)	0	0
	Encephalomyopathy/seizure	18 (36.7%)	9 (42.9%)	9 (32.1%)	0	0
	Diabetes mellitus	10 (20.4%)	0	10 (35.7%)	0	0
	Cardiomyopathy	4 (8.2%)	2 (9.5%)	2 (7.1%)	0	0
	Proteinuria	2 (4.1%)	1 (4.8%)	1 (3.6%)	0	0
	Severe fatty liver	1 (2%)	0	1 (3.6%)	0	0
	Anemia/cytopenia	0	0	0	3 (75%)	1
	Hypoglycemia	0	0	0	1 (25%)	0
Organ involvement during the follow-up period	Neurologic	41 (83.7%)	19 (90.5%)	22 (78.6%)	4 (100%)	1
	Endocrine	26 (53.1%)	9 (42.9%)	17 (60.7%)	3 (75%)	1
	Cardiac	20 (40.8%)	9 (42.9%)	11 (39.3%)	2 (50%)	1
	Renal	9 (18.4%)	4 (19%)	5 (17.9%)	2 (50%)	1
	SNHL	36 (73.5%)	17 (81%)	21 (75%)	2 (50%)	1

MELAS, mitochondrial encephalomyopathy, lactic acidosis, and stroke-like episodes; SNHL, sensorineural hearing loss

Four children were diagnosed with Pearson syndrome at a median age of 3 months (range, 0.1 − 2.8 years). Three patients presented with anemia, and one with recurrent hypoglycemia. One patient with KSS manifested myelodysplastic syndrome at the age of 2 months and received chemotherapy. Over time, the patient developed clinical features with ophthalmoplegia, cardiac conduction defects, and renal tubular disease, suggesting KSS. The diagnosis of retinitis pigmentosa could not be confirmed because of difficulties with cooperation during the retinal examination.

### Molecular characteristics of patients with mitochondrial disease

Among the 42 families with MELAS, the m.3243 A > G in *MTTL1* was the most common (39/42, 92.8%), followed by m.8573G > A in *MTATP6* (*n* = 1), m.3946G > A in *MTND1* (*n* = 1), and m.8363G > A in *MTTK* (*n* = 1). Of the 23 patients with mitochondrial DM, 22 (95.7%) harbored the *MTTL1* m.3243 A > G mutation, whereas one (4.3%) carried the *MTTK* m.8363G > A mutation. All five patients with papillary thyroid cancer harbored the *MTTL1* m.3243 A > G mutation. The patient with hypoparathyroidism also had the *MTTL1* m.3243 A > G mutation. Thirteen patients from 10 families with cardiomyopathy harbored three different mutations as follows: *MTTK* m.8363G > A (one family), *MTND1* m.3943G > A (one family), and *MTTL1* m.3243 A > G mutations (eight families). All nine patients with nephropathy had the *MTTL1* m.3243 A > G mutations.

All patients with Pearson syndrome and KSS had large gene deletions. Four patients with Pearson syndrome harbored four different deletions: m.9497_13734del, m.12113_14421del, m.9424_14840del, and m.8926_13321del. The patient with KSS harbored the m.8283_13792del.

### Endocrine dysfunctions and long-term outcomes of 49 patients with MELAS

**Fig. 1:**
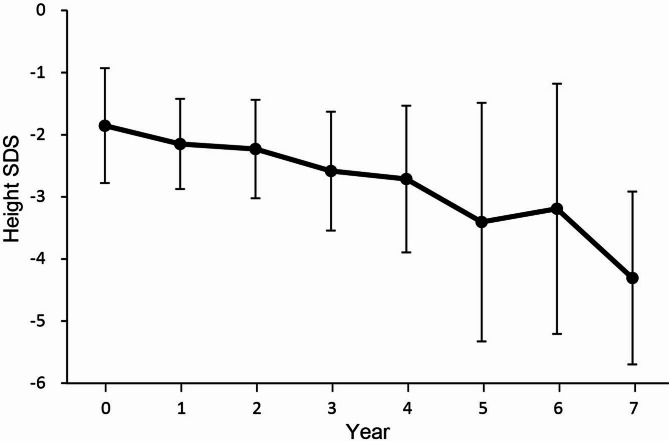
Trajectories of height SDS in patients with juvenile-onset MELAS

The endocrine characteristics of patients with MELAS are summarized in Table 2. The mean height and weight SDSs at diagnosis were − 2.0 ± 1.3 and − 2.6 ± 1.6, respectively, with a BMI SDS of − 1.6 ± 1.4. Among the patients, 29 reached final height, with height and weight SDSs of − 2.1 ± 1.5 and − 2.9 ± 1.4, respectively. Short stature was observed in 22 patients (44.9%) at diagnosis, with height and weight SDSs of − 3.1 ± 0.7 and − 3.4 ± 1.5, respectively. During the follow-up period, short stature was observed in 47.6% (*n* = 10) of pateints with juvenile-onset and 42.9% (*n* = 12) of patients with adult-onset. Figure 1 represents the longitudinal growth patterns of patients with MELAS who were followed up for at least 5 years during childhood and adolescence, demonstrating a progressive decline in height SDS over time.

**Table 2:** Endocrine characteristics of patients with MELAS

	Total (49)	Juvenile (21)	Adult (28)
Height SDS (mean ± SD)	–2.0 ± 1.3	–2.1 ± 1.5	–2.0 ± 1.3
Weight SDS (mean ± SD)	–2.6 ± 1.6	–2.2 ± 1.5	–3.1 ± 1.5
BMI SDS (mean ± SD)	–1.6 ± 1.4	–1.2 ± 1.6	–2.2 ± 1.2
Short stature at diagnosis, no. (%)	22 (44.9%)	10 (47.6%)	12 (42.9%)
Age at diagnosis (mean ± SD, years)	23.0 ± 17.7	8.0 ± 4.3	34.9 ± 14.6
Diabetes, no. (%)	23 (46.9%)	9 (42.9%)	14 (50%)
Median age at diagnosis (range)	26 (12, 50)	14.3 (12, 26)	35.5 (25, 50)
Median HbA1c (range)	7.9 (6.5, 18.5)	6.8 (6.5, 12.1)	9.5 (6.5, 18.5)
Initial treatment, no. (%)			
Life style modification	1 (4.3%)	1 (11.1%)	0
Insulin	12 (52.2%)	7 (77.7%)	5 (35.7%)
Sulfonylurea	5 (21.7%)	1 (11.1%)	4 (28.6%)
Metformin	5 (21.7%)	0	5 (35.7%)
Hypoparathyoidism, no. (%)	1 (2%)	0	1 (3.6%)
Age at diagnosis (years)	26	-	26
Calcium/Phosphorus (mg/dL)	7.5/4.8	-	7.2/4.8
PTH (pg/dL)	5.0	-	5.0
Papillary thyroid cancer, no. (%)	5 (10.2%)	0	5 (17.9%)
Age at diagnosis (mean ± SD, years)	34.1 ± 6.9	-	34.1 ± 6.9

BMI, body mass index; HbA1c, hemoglobin A1c; no., number; SD, standard deviation; SDS, standard deviation score

Twenty three patients with MELAS (46.9%) were diagnosed with mitochondrial DM at a median age of 26 years (range, 12 − 50 years). Among them, nine patients had juvenile-onset disease, whereas the remaining 14 had adult-onset disease. Nineteen patients had a family history of MIDD. The median hemoglobin A1c (HbA1c) level at diagnosis was 7.9% (range, 6.5 − 18.5%), and the random glucose level was 384.8 ± 292 mg/dL. Although most cases had an indolent onset, one patient presented with diabetic ketoacidosis at the age of 26 years. A total of 10 of 23 patients initially presented with DM and susequently developed additional neurological symptoms, leading to a diagnosis of MELAS at a median of 10 years after the onset of DM (range, 3 − 20 years). Five patients were initially treated with metformin; however, they were later switched to insulin therapy after their diagnosis of MELAS. The median duration of metformin treatment before the diagnosis of MELAS was 6 years (range, 1 − 10 years). The other patients have been treated with insulin injections.

**Fig. 2:**
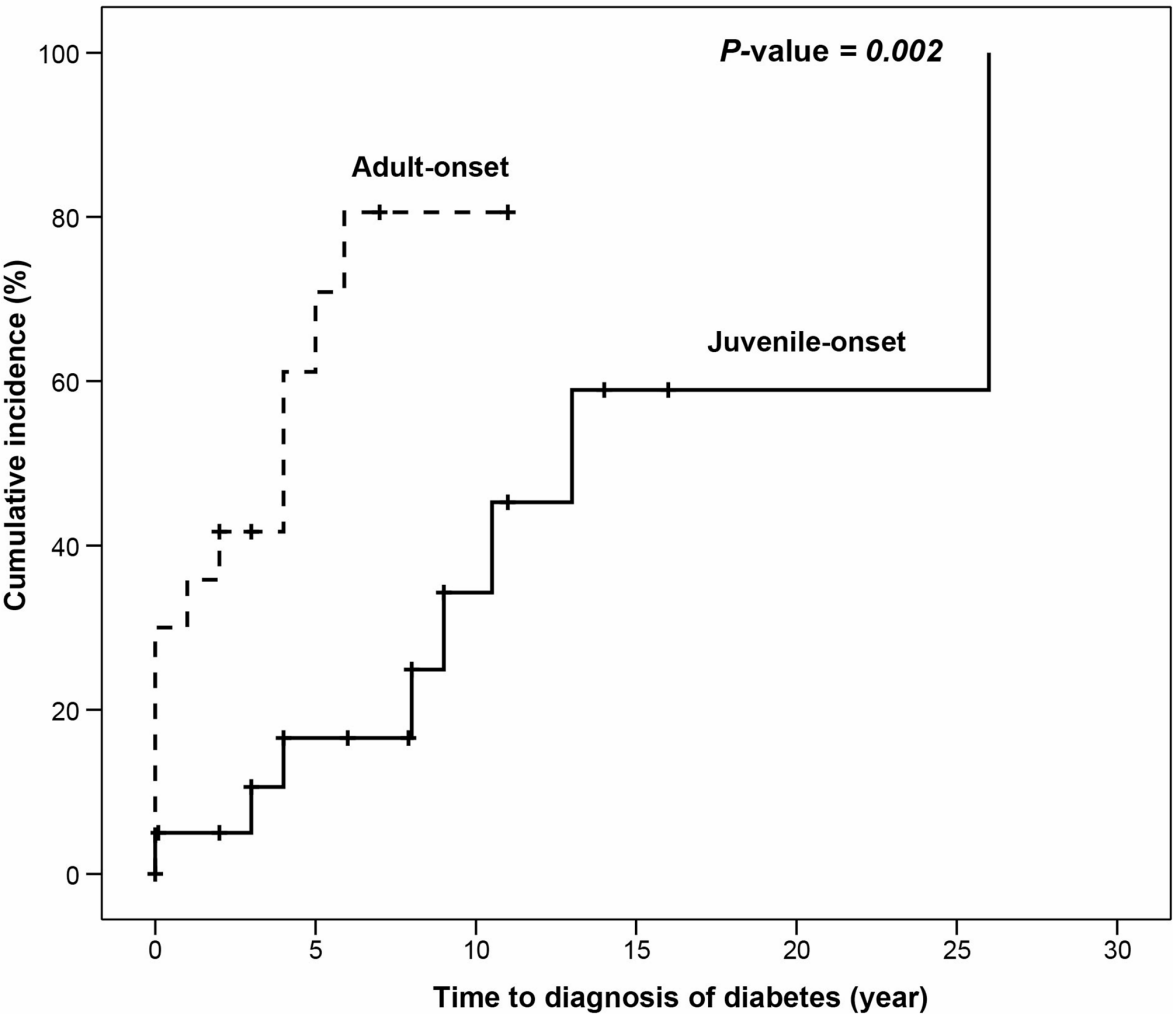
The Kaplan-Meier cumulative incidence curve for the time to diagnosis of diabetes in MELAS patients with the m.3243 A > G mutation

To further investigate factors influencing the onset of DM in MELAS patients with *MTTL1* mutations, we assessed associated risk factors during the follow-up period. Kaplan-Meier survival analysis revealed that adult patients with MELAS were either already diagnosed with DM at the time of diagnosis of MELAS or had a high likelihood of developing DM within 5 years (Fig. 2). In contrast, pediatric patients exhibited a gradual onset of DM following diagnosis of MELAS. Cox proportional hazards regression analysis was used to examine potential risk factors associated with diabetes onset in MELAS patients with *MTTL1* mutations. Gender, neurological symptoms, renal disease, and thyroid cancer were not significantly associated with the time to diabetes onset (Table 3). However, adult patients exhibited a significantly faster progression to diabetes compared to juvenile group (HR = 6.1, 95% CI: 1.76–21.1, *P* = 0.004).

**Table 3:** Multivariable Cox proportional hazard analysis of diabetes onset

	Diabetes onset		
	Hazard ratio (HR)	95% CI	*P* value
Sex			
Male	1.0		
Female	0.53	0.17–1.69	0.534
Group			
Juvenile	1.0		
Adult	6.1	1.76–21.1	0.004
Comorbidities			
Stroke	1.1	0.35–3.44	0.869
Renal involvement	1.0	0.33–3.3	0.958
Cardiac involvement	5.92	1.93–18.2	0.002
Thyroid cancer	2.37	0.57–9.87	0.233

Papillary thyroid cancer was diagnosed in 10.2% of patients (3 female and 2 male patients) at the mean age of 34.1 ± 6.9 years; all had adult-onset disease. Two patients were diagnosed with thyroid cancer concurrently with MELAS, whereas three were diagnosed with MELAS at a median of 9 years after their diagnosis of papillary thyroid cancer. Two of them also had DM. One patient had stage T1b N1b M0 cancer, and another patient had a larger tumor staged as T3 N0 M0 with capsular invasion; both required radioactive iodine therapy. The remaining three patients exhibited no evidence of lymph node or distant metastasis.

One patient presented with asymptomatic hypocalcemia with a total calcium level of 7.2 mg/dL and a low PTH level of 5.0 pg/mL (normal range, 10 − 65 pg/mL) and was consequently diagnosed with hypoparathyroidism. The patient has been treated with calcium and active vitamin D supplementation.

Two patients were diagnosed with MELAS after solid organ transplantation; one underwent heart transplantation and died 15 years later, whereas the other underwent kidney transplantation and is currently bed-ridden 4 years postoperatively.

### Endocrine dysfunctions and long-term outcomes of four patients with Pearson syndrome and one patient with KSS

The mean height and weight SDSs of patients with Pearson syndrome were − 1.6 ± 1.5 and − 1.4 ± 1.5, respectively, with a median age 0.3 months (range, 0.1 − 2.8 years) at diagnosis. Two patients died at 1 month and 11 months of age, respectively. The remaining two patients were followed up until they reached 15 years and 4.5 years of age, respectively. At the last follow-up, the height SDSs of the patients were − 7.5 and − 4.0, respectively, and the weight SDSs were − 6.5 and − 2.9, respectively.

Two patients with Pearson syndrome exhibited primary adrenal insufficiency. At 3.9 years of age, a hypoglycemic event occurred, and blood tests revealed serum glucose, adrenocorticotropic hormone (ACTH), and cortisol levels of 20 mg/dL, 3,890 pg/mL, and 16.8 µg/dL, respectively. The patient was subsequently treated with a physiologic dose of hydrocortisone. Owing to stunted growth velocity (< 2 cm/year), growth hormone (GH) stimulation tests were conducted using levodopa and regular insulin at 6.4 years of age, which demonstrated peak GH levels of 26.8 ng/mL and 37.2 ng/mL, respectively. The other patient with Pearson syndrome also experienced recurrent episodes of hyponatremia, hypokalemia, and hypoglycemia, with blood tests revealing ACTH and cortisol levels of 312 pg/mL and 15 µg/dL, respectively, at the age of 2.6 years, necessitating hydrocortisone treatment. Plasma renin activity and aldosterone level were 1.6 ng/mL/hr (normal range, 0.68 − 1.36) and 33.6 ng/dL (normal range, 3.57 − 24.0), respectively. The patient developed primary hypothyroidism at 3.4 years of age.

The patient with KSS manifested hypocalcemia (serum calcium level of 6.7 mg/dL) and a low PTH level of 3.0 pg/dL, consistent with hypoparathyroidism at 4 years of age. By 7 years of age, the patient exhibited stunted growth velocity (1 cm/year) with height and weight SDSs of − 5.2 and − 2.9, respectively, and developed chronic kidney disease. The serum IGF-1 level was 69 ng/mL (normal range, 90.6 − 265.9), and a GH stimulation test was not performed. After 1 year of recombinant human GH therapy, the growth velocity improved to 5.7 cm/year. However, GH therapy was discontinued because of decline in growth velocity to 2 cm/year after the second year of treatment.

## Discussion

This study demonstrated that patients with primary mitochondrial disease could manifest diverse endocrine dysfunctions, such as growth retardation, hypoparathyroidism, and mitochondrial DM, often accompanied by multiorgan involvement, particularly neurological abnormalities. Interestingly, papillary thyroid cancer was occasionally detected in patients with MELAS. In patients with Pearson syndrome and KSS, adrenal insufficiency and hypoparathyroidism were frequently observed during childhood.

Several factors contribute to growth retardation in early childhood including reduced oral intake due to dysphagia, vomiting, and neuromuscular manifestations [18, 19]. Mitochondrial dysfunction can also impair fetal growth, resulting in significantly small neonates at birth [20, 21]. Although the relationship between GH deficiency and mitochondrial disease remains unclear, GH deficiency has been reported in some cases [22]. In our study, short stature was observed in 48% of patients diagnosed before the age of 18 years and in 43% with adult-onset disease. At diagnosis, 45% of patients with MELAS in our study already exhibited short stature and experienced a progressive decline in height SDS over time. This trend suggests that systemic deterioration, such as poor nutrition and worsening of underlying disease, negatively impacted growth velocity. Therefore, mitochondrial diseases should be considered in patients presenting with short stature, low BMI, DM, and neurological features, such as developmental delay or hearing loss. Among five patients with Pearson syndrome and KSS, four exhibited short stature at the last follow-up, except for one patient who died at 1 month of age. These patients often had concomitant renal and cardiac dysfunction, resulting in poor outcomes.

The clinical features of mitochondrial DM are characterized by the absence of the typical features of type 1 or type 2 DM [23]. Patients with mitochondrial DM in adulthood often present with short stature and low BMI compared to patients with obesity commonly observed in type 2 DM. In this study, patients with MELAS showed a short and lean body composition. Mitochondrial DM can also be distinguished from other types of monogenic diabetes by the presence of multiorgan involvement and maternal transmission patterns. Our patients presented indolence at the time of DM diagnosis, which is consistent with previous studies [1, 23]. Regular screening can facilitate early detection and timely treatment, potentially preventing acute or chronic complications of DM in patients with juvenile-onset MELAS. In patients with the m.3243 A > G mutation, rapid progression to insulin dependence has been reported [1, 23]. Metformin and thiazolidinediones should be avoided in patients with mitochondrial disease, as these drugs inhibit respiratory chain complex I and can aggravate lactic acidosis [3]. Although sulfonylurea is traditionally recommended for type 2 DM, patients with mitochondrial diseases have a significant risk of hypoglycemia or cardiovascular toxicity [24]. Our study highlights distinct patterns of diabetes onset in MELAS patients with *MTTL1* mutations. Pediatric patients exhibited a gradual increase in diabetes onset, whereas adult patients either had pre-existing DM at the time of diagnosis of MELAS or developed it rapidly following the onset of neurological symptoms. This finding underscores the need for close monitoring for endocrine manifestations, particularly in adult patients, to facilitate early intervention.

In the present study, 10% of patients with MELAS were diagnosed with papillary thyroid cancer, all of whom had adult-onset MELAS. Somatic mutations in mtDNA have been identified in several human cancers including bladder, breast, colorectal, lung, and thyroid cancers [25–27]. Previous studies have demonstrated a higher prevalence of somatic variants in genes encoding respiratory chain complex I compared to normal controls [28, 29]. Furthermore, mutations in mtDNA, particularly the m.3244G > A and m.3242G > A mutations adjacent to the m.3243 A > G mutation, have been observed as recurrent hotspots in human tumors [30–32]. However, a nationwide study in Denmark investigating the risk of cancer in patients with mtDNA mutations including those with the m.3243 A > G mutation did not identify an increased cancer risk associated with this mutation, reporting a standardized incidence ratio (SIR) of 1.06 for any primary cancer and a specific SIR of 0.94 for the m.3243 A > G mutation [33]. Additionally, a single case of homoplasmic m.3243 A > G mutation has been reported in colorectal cancer [32]. The m.3243 A > G mutation was not observed in the studies using thyroid tissues [26, 29, 34]. Although our patients exhibited a relatively higher incidence of thyroid cancer, a direct association with MELAS syndrome remains challenging owing to the limited sample size. The potential heteroplasmic state in thyroid tissues may influence tumorigenesis in patients with MELAS with thyroid cancer. To validate these observations, epidemiologic studies need to robustly assess the association between mtDNA mutations and cancer risk.

In the current study, adrenal insufficiency was rare in children with mitochondrial diseases, with only two cases of Pearson syndrome. Hypoparathyroidism was observed in one patient with KSS and in one patient with MELAS. However, mitochondrial disease needs to be considered a potential cause of primary adrenal insufficiency and hypoparathyroidism as these are features of mitochondrial disease [35]. Impaired mitochondrial ATP production and oxidative stress likely contribute to the diminished capacity for adrenocortical hormones and PTH production [3]. Furthermore, these conditions more frequently manifest at an earlier age than MELAS and were often associated with substantial multiorgan involvement.

This study has several limitations. The single-center retrospective study design resulted in missing data including laboratory findings and variable duration of follow-up. Additionally, pubertal assessment and laboratory tests, such as IGF-1, IGFBP-3, and GH stimulation tests, were not consistently performed across all patients.

## Conclusions

Endocrine dysfunctions may precede the neurological manifestations of MELAS. Physicians should suspect underlying mitochondrial disease in patients presenting with endocrine abnormalities with multisystem involvement. Although a relatively high incidence of papillary thyroid cancer was observed in our study, further research should establish a potential association between thyroid cancer and MELAS.

## Electronic supplementary material

Below is the link to the electronic supplementary material.

Supplementary Material 1

## Data Availability

The data that support the findings of the study are not publically available due to privacy issues of study subjects.

## Abbreviations

ACTH: Adrenocorticotropic hormone
ATP: Adenosine triphosphate
BMI: Body mass index
DM: Diabetes mellitus
GH: Growth hormone
IGF-1: Insulin-like growth factor 1
IGFBP-3: Insulin-like growth factor binding protein 3
IRMA: Immunoradiometric assay
KSS: Kearns–Sayre syndrome
LHON: Leber’s hereditary optic neuropathy
MELAS: Mitochondrial encephalomyopathy, lactic acidosis, and stroke-like episodes
MIDD: Maternally inherited diabetes mellitus and deafness
mtDNA: Mitochondrial DNA
PCR: Polymerase chain reaction
PTH: Parathyroid hormone
SDS: Standard deviation score
SIR: Standardized incidence ratio
SNHL: Sensorineural hearing loss
T4: Thyroxine
TSH: Thyroid-stimulating hormone
RIA: Radioimmunoassay
