# Supplementary material for: Endocrine manifestations and long-term outcomes of patients with mitochondrial diseases
Source: Orphanet J Rare Dis. 2025 May 17;20:235. doi: 10.1186/s13023-025-03773-6 (PMC12085821; doi:10.1186/s13023-025-03773-6)
Supplement: Supplementary file 1 — Supplementary Material 1 [file 13023_2025_3773_MOESM1_ESM.docx]

**Table S1** Sequences of oligonucleotide primers for mitochondrial gene sequencing

| Positions | Sense (5′-to-3′) | Antisense (5′-to-3’ ′ | Size (bp) |
| --- | --- | --- | --- |
| m.591_1430 | cctcctcaaagcaatacactg | tgctaaatccaccttcgacc | 840 |
| m.1411_2026 | cgatcaacctcaccacctct | tggacaaccagctatcacca | 802 |
| m.1830_2688 | ggactaacccctataccttctgc | ggcaggtcaatttcactggt | 859 |
| m.2480_3365 | aaatcttaccccgcctgttt | aggaatgccattgcgattag | 886 |
| m.3150_3980 | tacttcacaaagcgccttcc | atgaagaatagggcgaaggg | 831 |
| m.3777_4679 | tggctcctttaacctctcca | aaggattatggatgcggttg | 903 |
| m.4466_5443 | actaattaatcccctggccc | aatggggtgggttttgtatg | 978 |
| m.5238_6031 | ctaaccggctttttgccc | acctagaaggttgcctggct | 813 |
| m.5835_6661 | gaggcctaacccctgtcttt | attccgaagcctggtaggat | 827 |
| m.6450_7334 | ctcttcgtctgatccgtcct | agcgaaggcttctcaaatca | 886 |
| m.7129_8114 | acgccaaaatccatttcact | cgggaattgcatctgttttt | 987 |
| m.7908_8816 | acgagtacaccgactacggc | tgggtggttggtgtaaatga | 909 |
| m.8062_9416 | tttccccctctattgatccc | gtggccttggtatgtgcttt | 815 |
| m.9211_10149 | cccaccaatcacatgcctat | tgtagccgttgagttgtggt | 939 |
| m.9967_10858 | tctccatctattgatgagggtct | aattaggctgtgggtggttg | 892 |
| m.10653_11531 | gccatactagtctttgccgc | ttgagaatgagtgtgaggcg | 859 |
| m.11295_12095 | tcactctcactgcccaagaa | ggagaatgggggataggtgt | 801 |
| m.11929_12793 | tatcactctcctacttacag | agaaggatataattcctacg | 865 |
| m.12551_13526 | aaacaacccagctctccctaa | tcgatgatgtggtctttgga | 976 |
| m.13319_14287 | acatctgtacccacgccttc | agaggggtcagggttgattc | 969 |
| m.14081_14988 | gcataattaaactttacttc | agaatattgaggcgccattg | 937 |
| m.14837_15997 | tgaaacttcggctcactcct | agctttgggtgctaatggtg | 1,162 |
| m.15792_31 | tcattggacaagtagcatcc | gagtggttaatagggtgatag | 810 |
| m.16401_794 | caccatcctccgtgaaatca | aggctaagcgttttgagctg | 964 |

Primers were designed with primer3 cgi v.2.0 served from Whitehead Institute (https://primer3.ut.ee/) using sequences from GenBank accession number of NC_012920.1.

**Table S2** Sequences of oligonucleotide primers for detecting mitochondrial gene deletions

| Positions | Sense (5′-to-3′) | Antisense (5′-to-3′) | Size (bp) |
| --- | --- | --- | --- |
| m.591_9397 | cctcctcaaagcaatacactg | gtggccttggtatgtgcttt | 8807 |
| m.2480_m.8095 | aaatcttaccccgcctgttt | cgggaattgcatctgttttt | 5616 |
| m.5385_m.15958 | gaggcctaacccctgtcttt | agctttgggtgctaatggtg | 7574 |
| m.7908_m.14268 | acgagtacaccgactacggc | agaggggtcagggttgattc | 6361 |
| m.9397_m.1430 | gtggccttggtatgtgcttt | tgctaaatccaccttcgacc | 8632 |
| m.14081_m.2688 | gcataattaaactttacttc | ggcaggtcaatttcactggt | 5176 |
| m.14837_m.6661 | tgaaacttcggctcactcct | attccgaagcctggtaggat | 8393 |
